# A simple phenol-free isolation method for high-quality RNA from bilberry

**DOI:** 10.1016/j.mex.2021.101481

**Published:** 2021-08-09

**Authors:** Kai Wang, Mengxia Liu, Fuqiang Cui, Fred Asiegbu

**Affiliations:** a Department of Forest Sciences, University of Helsinki, Helsinki, Finland; b State Key Laboratory of Subtropical Silviculture, Zhejiang A&F University, Lin'an, Hangzhou 311300, China

**Keywords:** RNA isolation, CTAB, Fruit pulp, Fruit peel, Bilberry, RIN

## Abstract

High-quality RNA is required for accurate gene expression and transcriptome analysis. The current methods of RNA extraction from berry fruits are either time-consuming or expensive. To simplify the conventional phenol-chloroform based RNA extraction method, we modified the protocol with less steps as well as the removal of the use of phenol. In this protocol, the extraction buffer is composed of hexadecyltrimethyl ammonium bromide (CTAB), polyvinylpyrrolidone (PVP), and Dithiothreitol (DTT). The method facilitates efficient removal of polysaccharides and phenolic compounds from both fruit pulp and fruit peel. Additionally, the protocol is phenol-free and less toxic than traditional phenol-containing method. High-quality RNA, with RNA Integrity Number value > 8, isolated by this method is applicable for RNA sequencing and qPCR. Only 3–4 working hours are required for one batch of RNA isolation.

• Our method replaces the use of phenol-chloroform with chloroform, making the extraction less toxic.
• The bilberry friut RNA is of high-quality and purity with less time input.

## Graphical abstract

**Figure:**
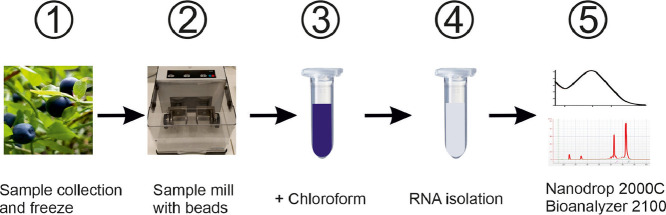

Specifications Table

**Table:** 

Subject Area:	Agricultural and Biological Sciences
More specific subject area:	Forestry
Method name:	A simple phenol-free isolation method for high-quality RNA from bilberry
Name and reference of original method:	Isolation of high quality RNA from bilberry (*Vaccinium myrtillus* L.) fruit Jaakola L, Pirttilä AM, Halonen M, Hohtola A. Isolation of high quality RNA from bilberry (*Vaccinium myrtillus* L.) fruit. Mol Biotechnol. 2001;19(2):201-3.
Resource availability:	NanoDrop 2000C: https://www.thermofisher.com/order/catalog/product/ND-2000#/ND-2000 Agilent 2100 bioanalyzer: https://www.agilent.com/en/product/automated-electrophoresis/bioanalyzer-systems/bioanalyzer-instrument Mixer Mill MM400 https://www.retsch.com/products/milling/ball-mills/mixer-mill-mm-400/function-features/

## Introduction

RNA isolation is a vital step to ensure data quality for RNA sequencing and transcriptomic study. Berry fruits are rich in polysaccharides and phenolic compounds, which affect the quality and/or quantity of isolated RNA [1,2]. Consequently, RNA isolation from fruits is challenging and time-consuming [3,4], compared to the method used for other plant tissues. Modified protocols have been developed with CTAB extraction buffer for bilberry and conifer trees [4], [5], [6]. However, we found a more simplified method to yield high-quality RNA from bilberry, with simpler steps and less time. In addition, chloroform: isoamyl alcohol (IAA) (24:1) was used instead of traditional phenol:chloroform:IAA (25:24:1), making the working environment less toxic.

## Materials and methods

Bilberry fruits were collected from wild-grown forestry (60 °20′33.1"N 25 °03′54.6"E) in Southern Finland, and were separated into pulp and peel, which were directly kept in liquid nitrogen and stored at -80°C until needed. Frozen pulp and peel samples were milled for 60 s at 22 Hz in a Mixer Mill MM400 (Retsch technology, Haan, Germany) in 2 ml Eppendorf tube with 5 mm sterile steel ball. Three replicates (∼100 mg/replicate) for both pulp and peel samples were applied.

The procedure details are as follows:

1. Add 900 µL preheated CTAB buffer (65 °C) and 9 μl DTT (1 mol/L) to each sample (∼80 mg powder in 2 ml Eppendorf tube) and vortex for 3 s. Incubate the tubes for 20 min at 65 °C with 1000 rpm shaking (Thermomixer comfort, Eppendorf).
2. Centrifuge the tubes at 11,000 rpm g for 10 min at room temperature.
3. Pipette the supernatants to new 2 ml tubes and extract twice with an equal volume of chloroform:IAA (24:1), separating the phases at 12,000 rpm for 10 min at room temperature.
4. Add 1/4 v of 10 M LiCl (42.4 g/mol) to the supernatant and mix gently. The RNA is precipitated overnight at + 4 °C.
5. Centrifuge the tubes at 13,000 rpm for 20 min at + 4 °C.
6. Wash the pellet with 500 µL 70% cold ethanol. Centrifuge the tubes 5 min and decant the ethanol. Dissolve the RNA pellets in 100 µL TE buffer.
7. Add 300 µL 100% cold ethanol and centrifuge 5 min. Dissolve the RNA pellets in 20 µL TE buffer.

Total RNA concentration was measured with NanoDrop 2000C and RNA integrity (RIN) was assessed with Agilent 2100 bioanalyzer based on manufacturer's instructions.

## Method validation

**Fig. 1:**
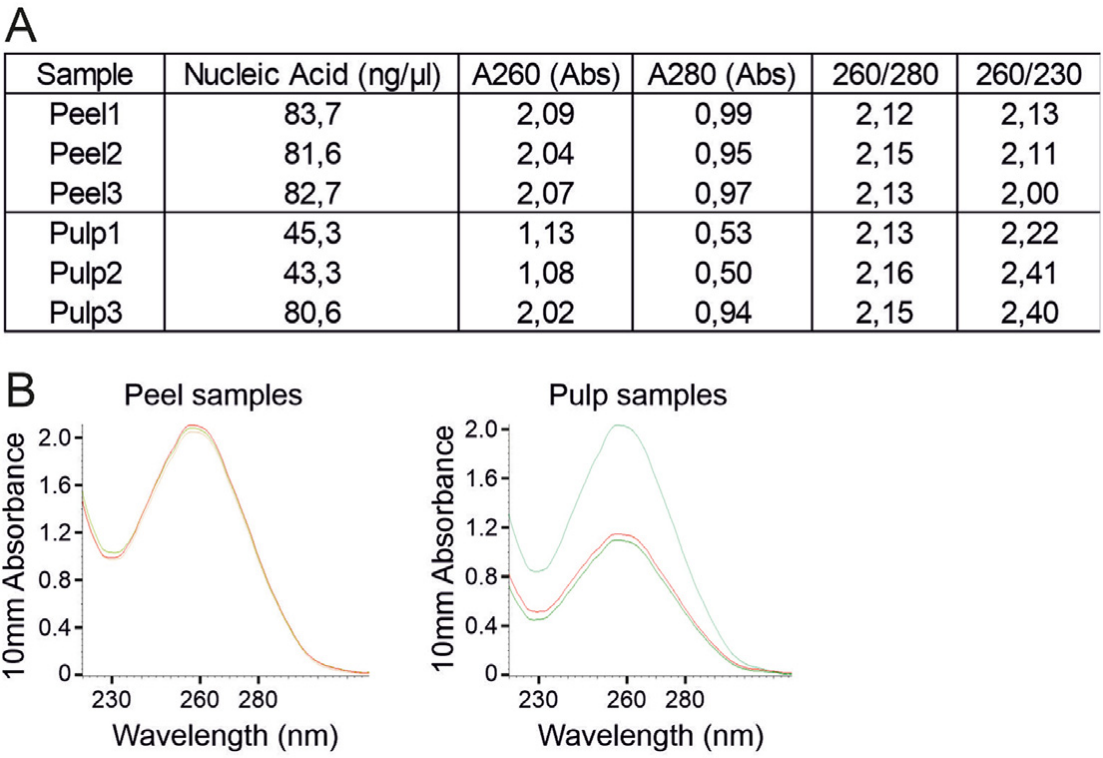
NanoDrop measurement of RNA samples. A), RNA concentration, 260/280 nm and 260/230 nm ratios. B), NanoDrop absorption spectra of RNA samples. Peak UV absorbance occurs at 260 nm.

**Fig. 2:**
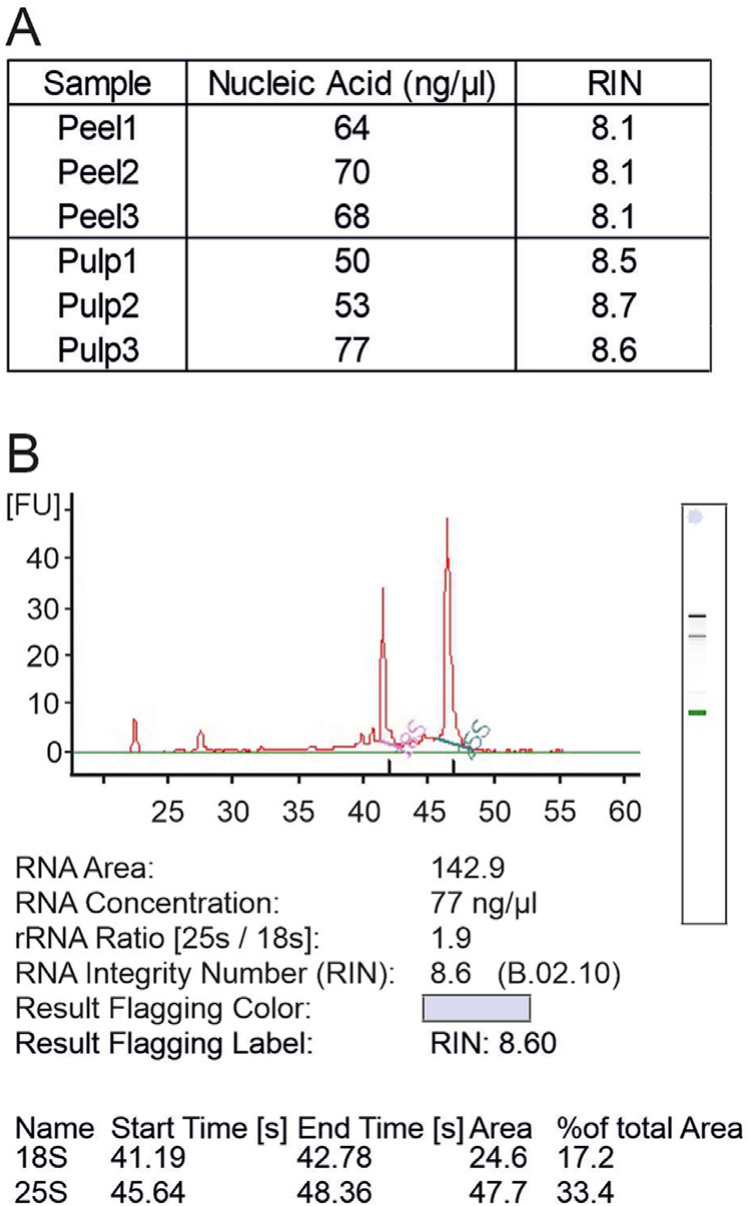
Bioanalyzer measurement of RNA samples. A), RNA concentration and RIN values. B), Agilent profile of a representative sample Pulp3.

Both pulp and peel RNA with high amount and high quality were harvested. RNA concentration measured with NanoDrop ranged from 43–84 ng/µl. The 260/280 nm and 260/230 nm ratios from NanoDrop ranged between 2.12–2.16 and 2.00–2.41 respectively (Fig 1, A). The 10mm absorbance of 6 samples were peaked at around 260 nm and plunged at about 230 nm (Fig 1, B). To describe the RNA quality more precisely, we performed bioanalyzer measurement. RNA concentration measured with bioanalyzer ranged between 50–77 ng/µl. The RNA integrity number [7] were between 8.1 and 8.7 (Fig 2, A). The RIN numbers indicated that all of isolated RNA samples were well integrated for downstream application, such as RNA sequencing and cDNA biosynthesis. The peaks of 18S and 25S rRNA were captured with electrophoretic separation, with sample Pulp3 as an example (Fig 2, B).

Compared to previously reported berry RNA isolation method [4], the advantages of this protocol include time-saving and simplified extraction steps. Specifically, we conducted chloroform:IAA wash only before LiCl RNA precipitation. Normally a second wash with phenol:chloroform was usually conducted to enhance the removal of proteins and DNA from fruit samples [4, 8]. However, the phenol:chloroform:IAA and chloroform:IAA washing steps (step 9–11) after LiCl precipitation [4] were not necessary in this method. Consequently, we saved 1–2.5 h that raised by additional washing and re-precipitation steps. Additionally, the use of phenol is not necessary, which reduces the reagent cost (0.3€/tube) and the associated risk of handling toxic chemicals. Careful pipetting in Step 3 is key to reduce the level of contamination, by leaving about 100 µL upper liquid layer. LiCl precipitation at + 4 °C overnight ensures the purity of precipitated RNA. The total RNA extraction procedures, based on our experience, usually takes 3–4 h for one isolation batch with 6 samples.

In this study, we performed two steps of ethanol washing (70% and absolute) to ensure the removal of salts and final RNA precipitation. We obtained about 1 µg total RNA from each 2ml Eppendorf tube. To gain more yield or a higher concentration, simple combination of multiple technique replicates after Step 4 is beneficial, as stated in previously reported method [4]. The contaminants were well removed from bilberry fruits and peels, which contain high proportion of phenolic compounds and carbohydrates.

In addition to bilberry, we equally successfully tested this protocol with spruce needles. The needle RNA concentration measured with NanoDrop was 109–885 ng/µl, with 260/280nm and 260/230 nm ratios that ranged between 1.93–2.13 and 1.70–2.34 respectively. Thus, this protocol could be extended to other plant materials such as spruce and pine needles. This method will significantly save time or cost, especially when performing large-scale RNA isolation from fruit tissues such as bilberry. In conclusion, we provide a simplified and effective RNA isolation method especially for fruits of bilberry and it can equally be used for conifer tissues like spruce and pine needles.
